# Ertapenem-induced Encephalopathy in a Patient With Systemic Lupus Erythematosus and Preserved Renal Function: A Diagnostic Challenge

**DOI:** 10.7759/cureus.91410

**Published:** 2025-09-01

**Authors:** Shreya Muralidharan, Elangovan Raman, Sorabh Sharma

**Affiliations:** 1 Internal medicine, Vydehi Institute of Medical Sciences and Research Centre, Bengaluru, IND; 2 Internal Medicine, Madras Medical College and Rajiv Gandhi Government General Hospital, Chennai, IND; 3 Internal Medicine, University of Arizona College of Medicine - Tucson, Tucson, USA

**Keywords:** acute reversible encephalopathy, ertapenem-induced encephelopathy, mdr bacteria infection, mdro ecoli cauisng uti, normal renal function

## Abstract

Carbapenem antibiotics are recognized for their efficacy against multidrug-resistant infections but are occasionally associated with neurotoxicity, particularly encephalopathy, predominantly in elderly patients with renal impairment. We report a case of an elderly female patient with systemic lupus erythematosus (SLE), hypertension, and preserved renal function who developed encephalopathy shortly after initiation of ertapenem for a multidrug-resistant *Escherichia coli* urinary tract infection. The patient manifested vivid hallucinations and confusion, prompting an extensive evaluation that ruled out infectious, metabolic, and autoimmune etiologies. Discontinuation of ertapenem alongside empiric intravenous thiamine resulted in rapid and marked clinical improvement. This case underscores the potential for ertapenem-induced neurotoxicity even in the absence of renal dysfunction.

## Introduction

Ertapenem is a broad-spectrum carbapenem antibiotic frequently employed as monotherapy for complicated intra-abdominal infections, pelvic infections, skin and soft tissue infections, urinary tract infections (UTIs), and community-acquired pneumonia [1]. Though generally well tolerated, neurotoxicity - including encephalopathy, hallucinations, agitation, and seizures - has been documented, particularly among elderly patients and those with impaired renal function [2].

Encephalopathy refers to a diffuse brain dysfunction resulting in altered consciousness, cognitive deficits, and behavioral changes. Ertapenem-induced encephalopathy is infrequent, especially in patients with preserved renal function. The incidence of carbapenem-associated neurotoxicity has been estimated to range between 3% and 11%, varying across different agents, with imipenem carrying the highest risk [3,4]. Established risk factors include advanced age, renal impairment, pre-existing central nervous system disease, and concurrent use of drugs that lower the seizure threshold [5]. Although ertapenem is generally considered safer compared to other carbapenems, recent case reports indicate that neurotoxicity can occur even in the absence of renal dysfunction [2,6,7].

Herein, we describe the case of a 64-year-old woman with a history of systemic lupus erythematosus and hypertension who developed acute encephalopathy shortly after initiation of ertapenem therapy for a multidrug-resistant urinary tract infection. Her clinical course was notable for progressive neuropsychiatric symptoms, including confusion and vivid hallucinations, which were temporally associated with the antibiotic exposure. Importantly, these symptoms resolved rapidly following discontinuation of ertapenem, supporting a diagnosis of drug-induced encephalopathy.

## Case presentation

A 64-year-old woman with a medical history significant for systemic lupus erythematosus and hypertension presented with fatigue, lightheadedness, and multiple episodes of diarrhea following recent antibiotic therapy for a urinary tract infection (UTI). On examination, she exhibited tachycardia and hypertension. Initial management included intravenous ceftriaxone and oral vancomycin for presumed UTI and *Clostridioides difficile* infection, supported by a positive *C. difficile* antigen test.

Subsequent urine culture grew multidrug-resistant *Escherichia coli*, leading to escalation to intravenous ertapenem. Soon after, the patient developed hypertensive urgency that necessitated intensive care unit admission and intravenous antihypertensive therapy. About 72 hours following the initiation of ertapenem, she began to experience worsening mental status with progressive confusion, striking visual hallucinations (such as imagining drinking soup or believing she was on a train), and markedly disorganized behavior.

**Figure 1:**
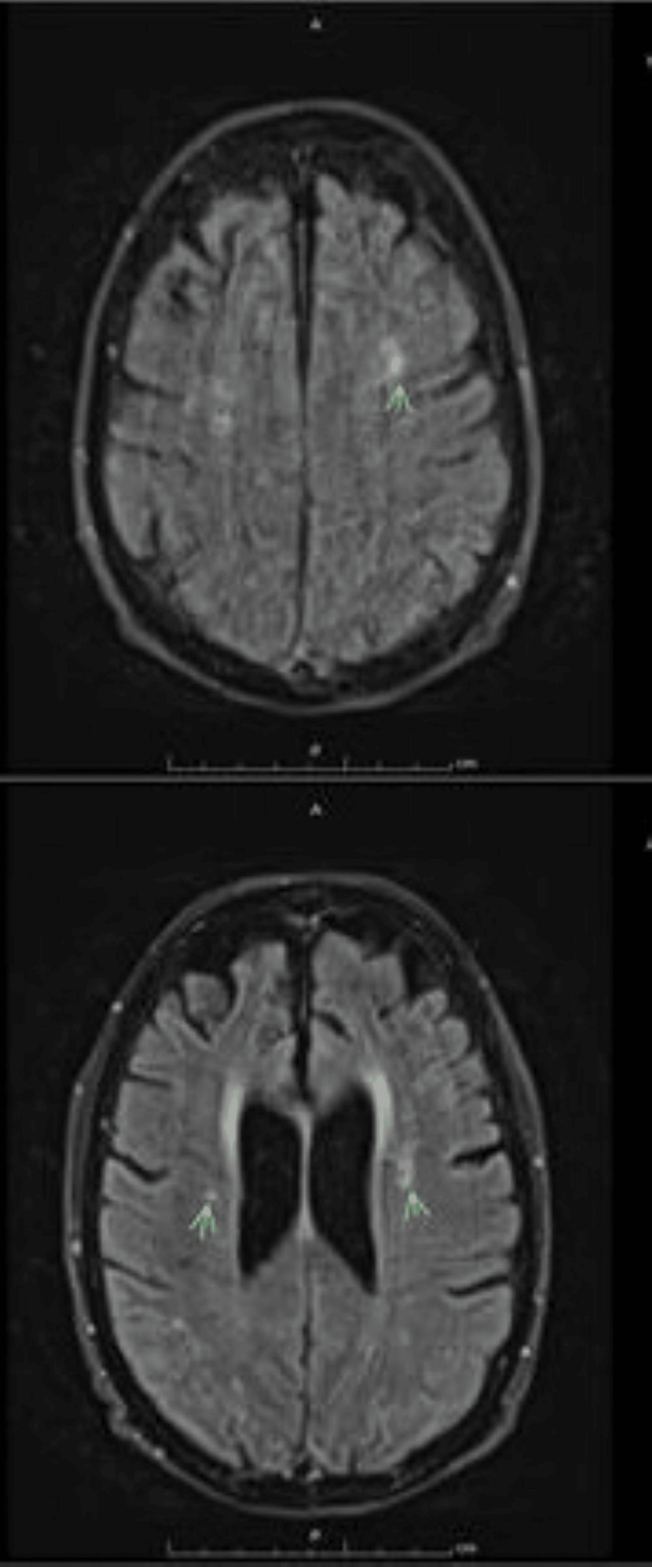
MRI - T2/fluid-attenuated inversion recovery (FLAIR) images demonstrate scattered supratentorial white matter hyperintensities compatible with chronic microvascular alterations. There is also diffuse cerebral volume loss with sulcal prominence. These changes are benign and doesn't explain the encephalopathy.

Diagnostic imaging, including brain MRI scan, showed no acute abnormalities (Figure 1). A comprehensive laboratory and toxicology workup, including urine drug screening, metabolic panels, and infectious markers, was unrevealing. Rheumatology and neurology consultations excluded lupus cerebritis. Laboratory investigations revealed mild hepatic transaminitis and urinary findings consistent with infection, as summarized in Tables 1, 2.

**Table 1:** Urinalysis Findings WBC: White blood cells; RBC: red blood cells.

Test	Result	Reference Range
Leukocyte Esterase	Positive	Negative
Nitrites	Positive	Negative
Urine White Blood Cells (WBCs)	Positive	0–5 cells/HPF
Urine Red Blood Cells (RBCs)	Positive	0–3 cells/HPF

**Table 2:** Liver Function Tests AST: Aspartate aminotransferase; ALT: alanine transaminase.

Test	Result	Reference Range (U/L)
AST	102 U/L	10–40 U/L
ALT	58 U/L	7–56 U/L

Due to the strong temporal association between ertapenem initiation and neuropsychiatric symptoms, ertapenem was discontinued. Empiric intravenous thiamine was administered to address the possibility of Wernicke’s encephalopathy, given the patient’s history of chronic alcohol use. The patient’s mental status markedly improved within 48 hours of ertapenem cessation, with full cognitive recovery prior to discharge in stable condition.

## Discussion

Carbapenem-associated neurotoxicity is an uncommon but established adverse event, with imipenem traditionally associated with the highest neurotoxic potential due to its gamma-aminobutyric acid (GABA)-A receptor antagonism [3,4]. Ertapenem, while generally safer, has increasingly been implicated in adverse effects of the central nervous system, even among patients with preserved renal function [5]. The proposed mechanism involves inhibition of GABA-A receptors, resulting in neuronal hyperexcitability and altered sensorium [6].

The underlying autoimmune condition, systemic lupus erythematosus, may have contributed to blood-brain barrier disruption and central nervous system immune dysregulation, thereby predisposing this patient to heightened neurotoxic effects. Similar instances of ertapenem-associated encephalopathy in patients without renal dysfunction have been documented in the literature, including a recent report by Khan et al., which further emphasizes that carbapenem-induced neurotoxicity can occur independently of renal impairment [7].

The differential diagnosis of acute encephalopathy is broad. Neuroleptic malignant syndrome (NMS) is an important mimic, characterized by altered mental status, muscular rigidity, fever, and autonomic instability. Absence of dopamine antagonist exposure and rigidity in this patient made neuroleptic malignant syndrome (NMS) unlikely. As highlighted by Raman et al. (2025), prompt exclusion of mimics like NMS is crucial to avoid delays in diagnosis of atypical encephalopathy [8].

The close temporal association between ertapenem administration and symptom onset, combined with rapid symptom resolution upon drug discontinuation, strongly supports Ertapenem-induced neurotoxicity as the etiology. Although intravenous thiamine may have contributed to recovery given the patient’s alcohol use history, the timing favors ertapenem withdrawal as the key therapeutic intervention.

## Conclusions

This case adds to the accumulating evidence that ertapenem-induced neurotoxicity can manifest even in patients with intact renal function, particularly among immunocompromised individuals such as those with systemic lupus erythematosus. Clinicians should maintain vigilance for drug-induced encephalopathy in patients presenting with acute neuropsychiatric symptoms during carbapenem therapy. Early recognition and cessation of the offending agent are critical to ensure complete neurological recovery and prevent unnecessary diagnostic procedures.
